# “We threw away the stones”: a mixed method evaluation of a simple cookstove intervention in Malawi

**DOI:** 10.12688/wellcomeopenres.17544.1

**Published:** 2022-02-14

**Authors:** Sepeedeh Saleh, Henry Sambakunsi, Debora Makina, Moses Kumwenda, Jamie Rylance, Martha Chinouya, Kevin Mortimer

**Affiliations:** 1 Department of International Public Health, Liverpool School of Tropical Medicine, Liverpool, L3 5QA, UK; 2 Malawi-Liverpool Wellcome trust Clinical Research Programme, Blantyre, Malawi

**Keywords:** Air pollution, particulate matter, PM2.5, improved stove, intervention, low- and middle-income countries

## Abstract

**Background:** Exposure to air pollution is responsible for a substantial burden of respiratory disease globally. Household air pollution from cooking using biomass is a major contributor to overall exposure in rural low-income settings. Previous research in Malawi has revealed how precarity and food insecurity shape individuals’ daily experiences, contributing to perceptions of health. Aiming to avoid a mismatch between research intervention and local context, we introduced a simple cookstove intervention in rural Malawi, analysing change in fine particulate matter (PM _2.5_) exposures, and community perceptions.

**Methods: **Following a period of baseline ethnographic research, we distributed *‘chitetezo mbaula’*, locally made clay cookstoves, to all households (n=300) in a rural Malawian village. Evaluation incorporated village-wide participant observation and concurrent exposure monitoring using portable PM _2.5_ monitors at baseline and follow-up (three months post-intervention). Qualitative data were thematically analysed. Quantitative analysis of exposure data included pre-post intervention comparisons, with datapoints divided into cooking and non-cooking (‘baseline’) periods. Findings were integrated at the interpretation stage, using a convergent design mode of synthesis.

**Results: **Individual exposure monitoring pre- and post-cookstove intervention  involved a sample of 18 participants (15 female; mean age 43). Post-intervention PM _2.5_ exposures (median 9.9μg/m ^3^ [interquartile range: 2.2–46.5]) were not significantly different to pre-intervention (11.8μg/m ^3^ [3.8–44.4]); p=0.71. On analysis by activity, ‘baseline’ exposures were found to be reduced post-intervention (from 8.2μg/m ^3^ [2.5–22.0] to 4.6μg/m ^3^ [1.0–12.6]; p=0.01). Stoves were well-liked and widely used by residents as substitutes for previous cooking methods (mainly three-stone fires). Most cited benefits related to fuel saving and shorter cooking times.

**Conclusions: **The cookstove intervention had no impact on cooking-related PM _2.5_ exposures. A significant reduction in baseline exposures may relate to reduced smouldering emissions. Uptake and continued use of the stoves was high amongst community members, who preferred using the stoves to cooking over open fires.

## Introduction

Air pollution – and fine particulate matter (PM _2.5_) in particular – is a widely recognised risk factor for cardiorespiratory and wider systemic disease, and the interactions between airborne particulates and climate change also have repercussions for health ^1– 3^. In Malawi, which is largely rural, air pollution is a persisting problem, stemming mainly from domestic cooking: Malawian households cook on average three times per day, using biomass fuel (usually firewood) on three stone fires ^4^.

Recent ethnographic work on ‘smoke’ in the Malawian setting highlighted the ways in which local experiences and values – often very different from those of western researchers – can shape locally-relevant priorities for intervention, and contextualised approaches ^4^. Centring local perspectives in this way, as well as constituting arguably the ‘right’ approach to global health problems, can optimise the suitability and sustainability of any subsequent solutions ^5– 7^.

In rural Malawi, where experiences of precarity, scarcity and food insecurity are common, these contextual realities often take precedence over externally proposed agendas such as ours. In a recent study exploring Malawian communities’ perceptions of health within a trial of advanced cookstoves ^8^, participants linked good health primarily to food security ^9^. Thus the research imperative in such contexts should be for cleaner air solutions which avoid amplifying existing daily challenges for residents, as well as appropriately addressing shared concerns. In considering options for cleaner cooking in LMICs such as Malawi, economic affordability for the majority is a key consideration ^10– 13^. Whilst initial costs of clean stoves are important here, also relevant are costs of ongoing fuel purchase, and maintenance and repair costs of any newly introduced technologies ^14– 17^.

Perceptions of the benefits of new technologies are also context specific. Studies set in various LMIC settings have cited flexibility, in terms of fuel use or place of cooking ^18, 19^, and ability to cook quickly or for large numbers of people ^12, 20, 21^ as important considerations. Whilst cleaner burning biomass-fuelled cookstoves have been largely rejected by health researchers due to suboptimal emission reductions, features such as more efficient fuel use are themselves highly valued by local populations, with consequent potential environmental impacts conferring additional advantage ^22^. Thus, while individual household interventions will not be sufficient to achieve clinically impactful reductions in PM _2.5_^23, 24^ there may be benefits to community-level adoption of locally relevant cleaner stove types in low-income settings such as Malawi. This could represent a useful interim step on the way to the much-needed provision of clean fuels at scale ^25^.

Following an extended period of ethnographic and monitoring groundwork in a village in Malawi ^4^, we provided locally-made clay stoves to every household. Realist evaluation aimed to assess residents’ views of the cookstoves as well as any changes in personal PM _2.5_ exposures three months after cookstove distribution.

## Methods

### Ethical considerations

The study was approved and sponsored by the LSTM Research Ethics Committee (20-022). In-country ethical approval was granted by the College of Medicine Research Ethics Committee (COMREC) in Blantyre (P.06/20/3069). Informed consent processed were completed for all participants involved in air quality monitoring. For other village residents, an extended process of community consent and introduction was undertaken, with engagement throughout the project ensuring continued consent for participation ^1^.

### Study setting and population

The study was set in a rural village of approximately 300 households in Southern Malawi: the site of previous ethnographic and baseline monitoring work ^4^. Cooking, mostly carried out by female household members on three-stone fires, constituted the main source of PM _2.5_ exposure in this setting ^26^. Further contextual details are as previously reported ^4^. All households in the village were involved in the participant observation work and the intervention, and in qualitative elements of the evaluation. For exposure monitoring, consenting adult participants were recruited with an aim of achieving a broadly representative sample of village residents, including both men and women, members of different household sizes and structures, and varied cooking needs. These participants had to be resident in the village and habitually spending six or more days per week in the village setting. Children (aged under 18) were not included.

### Study design and intervention

This was a before-after study. Following a period of extended participant observation around the village and individual baseline exposure monitoring in a total of 23 residents (between January and March 2020), all households in the village were given a locally produced firewood cookstove, or *chitetezo mbaula*, meaning ‘protecting stove’. These are moulded, natural-draught cookstoves made of clay, promoted by the nongovernmental organisation (NGO) sector in the region ^27^, and recently piloted in rural Malawi in advance of a large cookstove trial ^28^.

The cookstoves were introduced to key local representatives (including the chief and a local health surveillance assistant) at a small village meeting, with explanations of their use and some expected benefits, before distribution – without cost – to households. Although they were known about by many in the village, few households already owned one of these firewood cookstoves.

**Figure 1.**
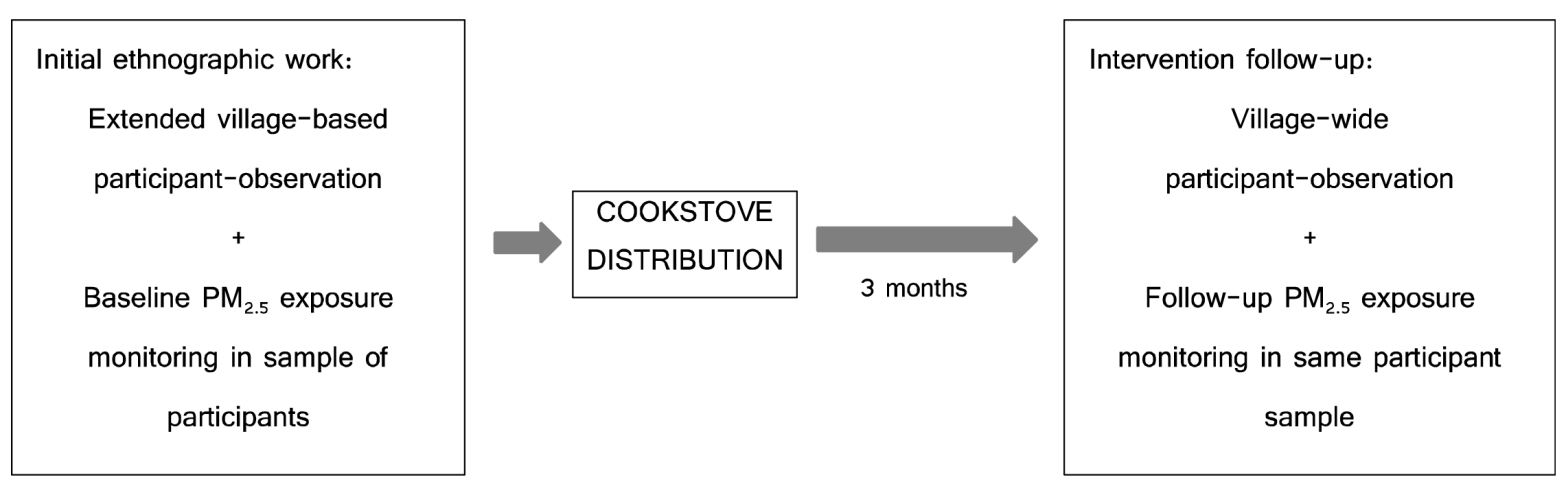
Visual depiction of study flow and combination of methods.

After three months, researchers (PhD research candidate, SS, and research assistant, HS) returned to the village and continued participant observations around the village. The originally sampled 23 residents were approached again for involvement in repeat PM _2.5_ exposure monitoring (March-April 2021) during the same evaluation period. These methods are depicted in Figure 1 below.

### Data collection

***Quantitative data collection.*** The original sample of 23 participants who took part in air pollution exposure monitoring were asked to each spend a further period of 48 hours carrying personal air quality monitors to assess post-intervention PM _2.5_ exposures. PurpleAir PA-II-SD laser particle counting devices (Purple Air, UT, USA) were used, as in the pre-intervention phase, again with 20Ah portable power banks (Anker Innovations, Changsha, China), and carried in specially designed waist bags. The devices took PM _2.5_ readings at two-minute intervals throughout the monitoring period.

As in the baseline study ^26^, on monitor collection, memory cards were removed and the data used to create simple line graphs on a laptop, which were then viewed together, by the participant and researcher, and used as a basis for activity recall. This technique (developed on the basis of earlier work using monitoring alongside participant observations), allowed for division of all traces into periods of ‘baseline’ (no identified exposure) and ‘activity’ (where a specific source of combustion was identified). Further information was gathered around each identified episode of cooking, including bathwater warming or fire/stove use for heating, place of cooking, stove or device, and fuel used ^29^.

***Qualitative data collection.*** Participant observations were carried out by the doctoral researcher (SS) and Malawian research assistant (HS), together with a local fieldworker: a village resident, and centred around cooking activity. As researchers and village residents were familiar with each other, following the initial period of ethnographic participant observation, observations were now spread around the village without the prior focus on a small number of individual households. Researchers visited the village on most days each week over a period of 10 weeks, spending time in all areas of the village over this observation period. Participant observation at this stage involved less active involvement by researchers in daily activities and more passive observation and discussion. Observations were mainly focused around evidence of stoves, fires, food, and fuel use.

Discussions were often based around cooking activities, partly because this was the activity families were most often engaged in when spending time around the household. These discussions were in reality more unstructured, participant-led conversations, and mainly concerned cooking and stove use, although other related topics were incorporated as was felt relevant by participants and researchers. Ad hoc conversations were held with any willing community members who were present at the time of our visits (although care and attention was always given to ethical issues including questions of confidentiality). In view of the social nature of the village setting, these conversations at times involved several women: either from an extended family group, or a group of village residents. At other times conversations were held with individual men and women. Conversations usually took place at residents’ homes, almost always outside houses, in yards or verandas. Contemporaneous field notes were made during this fieldwork, integrating discussion content and observations.

### Data analysis

***Analysis of PM _2.5_ exposure data.*** Descriptive comparisons of proportion of recorded time (datapoints) spent cooking, and specific cooking features (place, device and fuel used) before and after stove introduction were produced. Exposures before and after introduction of the stoves were compared using median and interquartile range values. Following division of all exposure datapoints into ‘activity’ or ‘baseline’, categories using matched time-activity data, medians and interquartile ranges before and after intervention introduction were compared for both ‘baseline’ and ‘activity’ subcategories. For statistical comparisons of pre- and post-intervention exposures, median exposures for each participant (pre- vs post-intervention) were compared using a Wilcoxon signed-rank test. A non-parametric test was chosen as the data did not consistently show a normal distribution ^30^. Data were analysed using R ^31^, and the package ggplot2 ^32^ was used to create plots.

***Analysis of participant observation data.*** Fieldnotes were jointly reviewed and reflected on by SS and HS with input from the local fieldworker, and tentative themes iteratively developed through these discussions. Content of the notes was entered onto QSR NVivo V.12 (released in March 2020) for formal coding (SS) and review (HS). The combination of participant observations with personal monitoring allows a number of benefits including triangulation – avoiding a reliance on ‘self-report’ by participants – and introducing insights into how interventions work within social contexts ^33^: particularly important in the case an intervention centred so firmly in the domestic sphere.

The combination of qualitative and quantitative enquiry, with each applied as appropriate, was used here as it allows for a fuller exploration of outcomes, particularly important for complex interventions with social elements ^34, 35^. Rather than separate but parallel applications and analysis, an integrated synthesis was used, allowing for more in-depth findings than when either single methodology is used alone. Qualitative and quantitative data collection were undertaken concurrently by the same research team, with integration happening at the interpretation stage: the so-called ‘Convergent Design’ model of mixed method study design ^36^.

## Results post-intervention

Between February 2020 and April 2021, 18 participants (15 female; mean age 43, standard deviation 14.2) completed the study with matching pre- and post-intervention traces (February – March 2020, and March – April 2021 respectively). The predominance of women in the sample reflected the majority female nature of cooking in the village. Three participants were lost from the full pre-intervention monitoring set (originally 23 participants) due to participants moving away from village (N=2) and participant death (N=1), and problems with monitors and batteries left only 18 with matching traces. The overall pre- and post-intervention dataset incorporated 1563 hours monitoring time (of which 788 hours post-intervention). In the pre-intervention dataset, trace lengths ranged from 23.3 to 58.5 hours (median 43.1; IQR 39.3 – 49.2). Post-intervention traces ranged between 24.1 and 53.9 hours (median 48.6; IQR 40.7 – 49.1). Traces shorter than 48H were due to battery faults.

Of the total recorded period (pre- and post-intervention), 351 hours (22.5%) constituted ‘activity’, of which 92% was cooking (including bathwater warming) activity. Other non-cooking activities included exposure to others’ fires or stoves (such as when socialising at a neighbour’s household), and burning grass on farmland. A larger proportion of the total post-intervention monitoring period constituted combustion activity compared with pre-intervention (30% post- vs. 23% pre-intervention). Further details are available on Harvard Dataverse ^29^.

### Cooking characteristics

**Figure 2.**
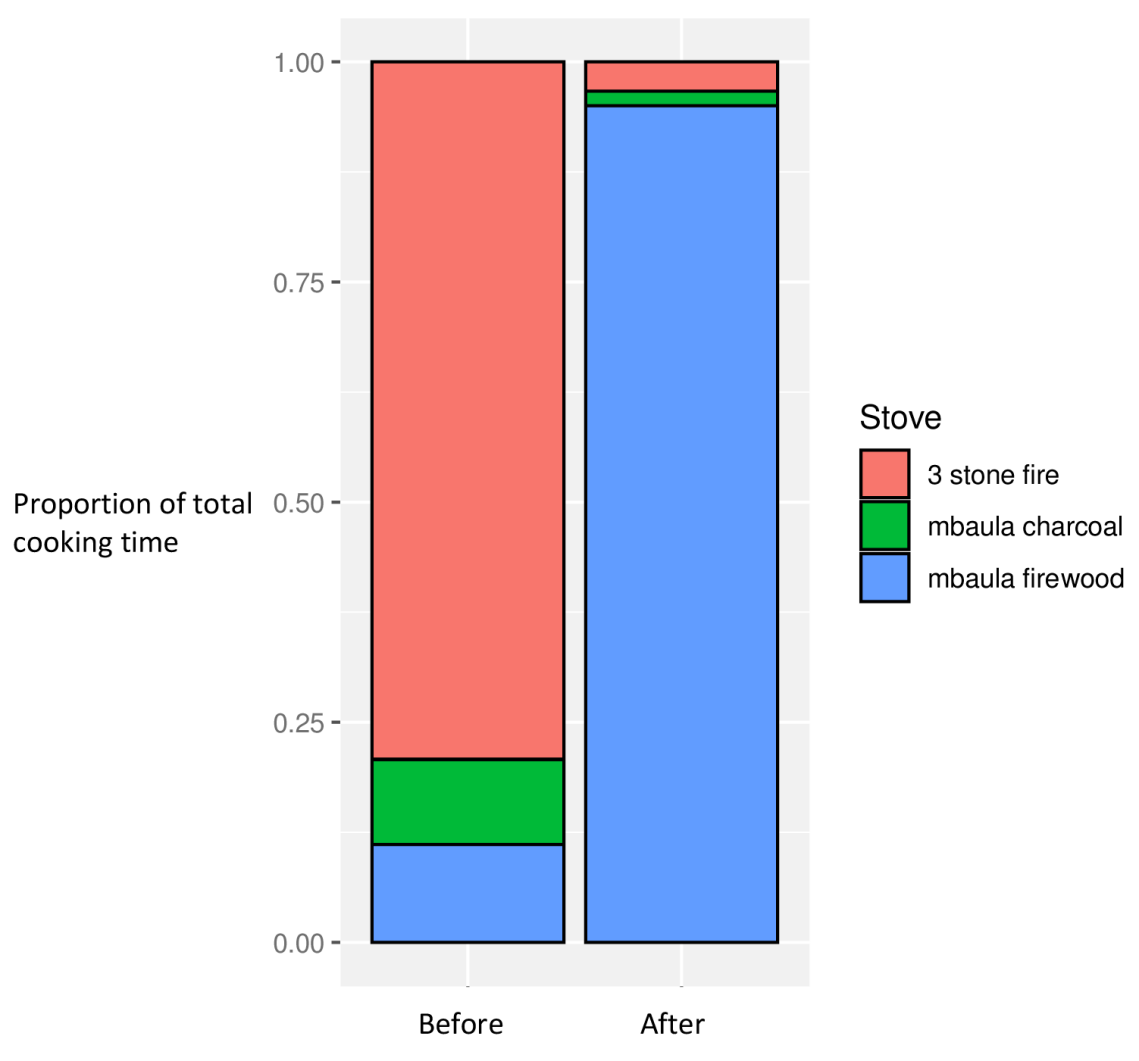
Proportion of overall cooking time by stove use, before and after intervention introduction.

In the baseline dataset, a majority of time spent cooking (across the dataset) employed three stone fires, with the remaining less than 20% of the time spent using charcoal or firewood stoves. After introduction of the firewood cookstoves to all households, over 95% of the overall cooking time was spent using the new stoves, with consequent reductions in use of three stone fires and charcoal stoves, now together constituting less than 5% of total cooking time ^29^ ( Figure 2).

**Figure 3.**
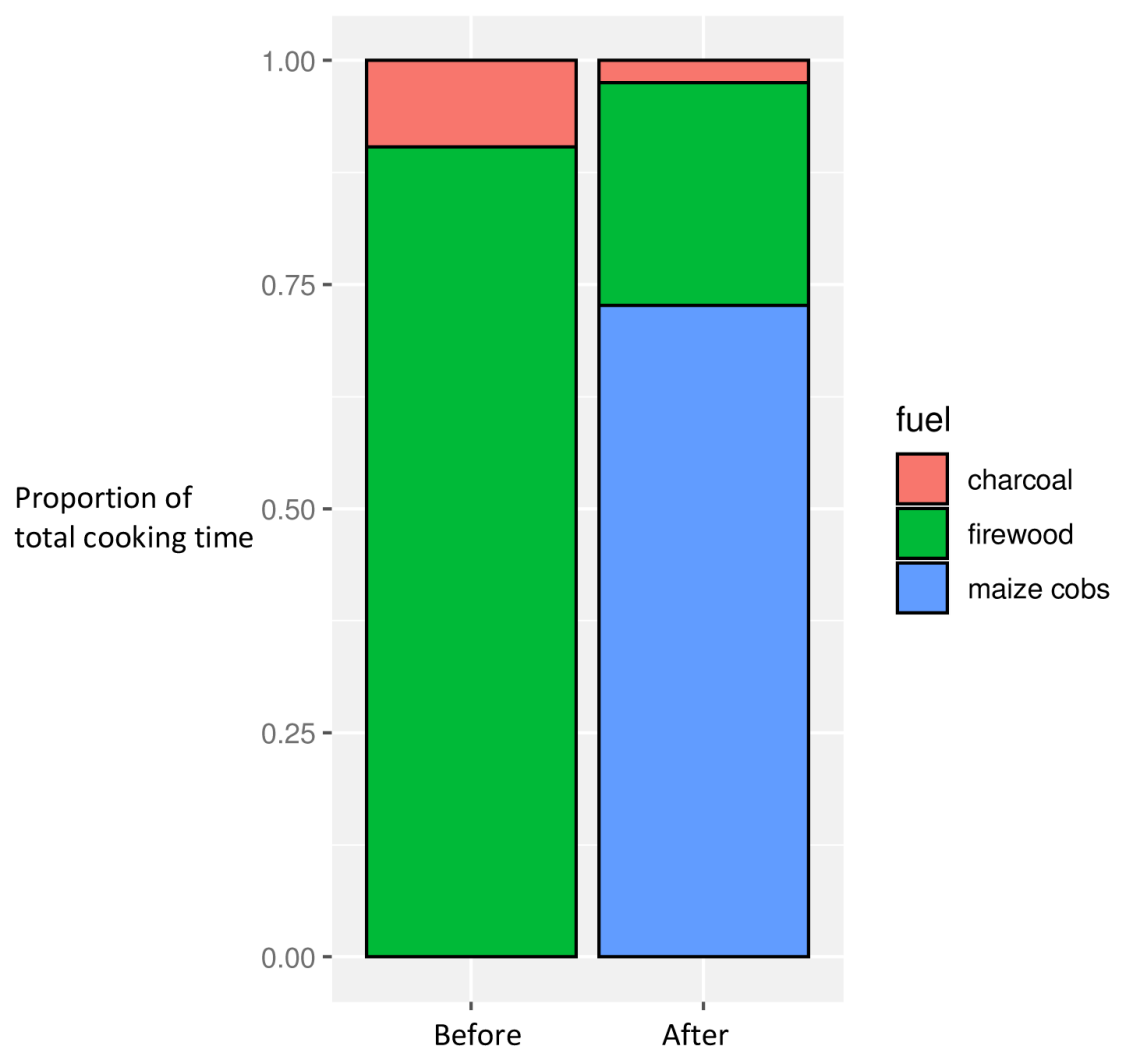
Proportion of overall cooking time by fuel use, before and after intervention introduction.

There were significant differences in fuel use in the before and after phases, with maize cobs widely used (in all but three households) post-intervention ( Figure 3). This was linked to the timing of the harvest: whilst pre- and post-intervention periods occurred at a similar time of year, the post-intervention phase coincided with the immediate post-harvest period such that maize cobs were freely available in the village and tended to be used as fuel in preference to other available fuel types such as wood and charcoal ^29^.

### PM _2.5_ concentrations before and after cookstove introduction

**Figure 4.**
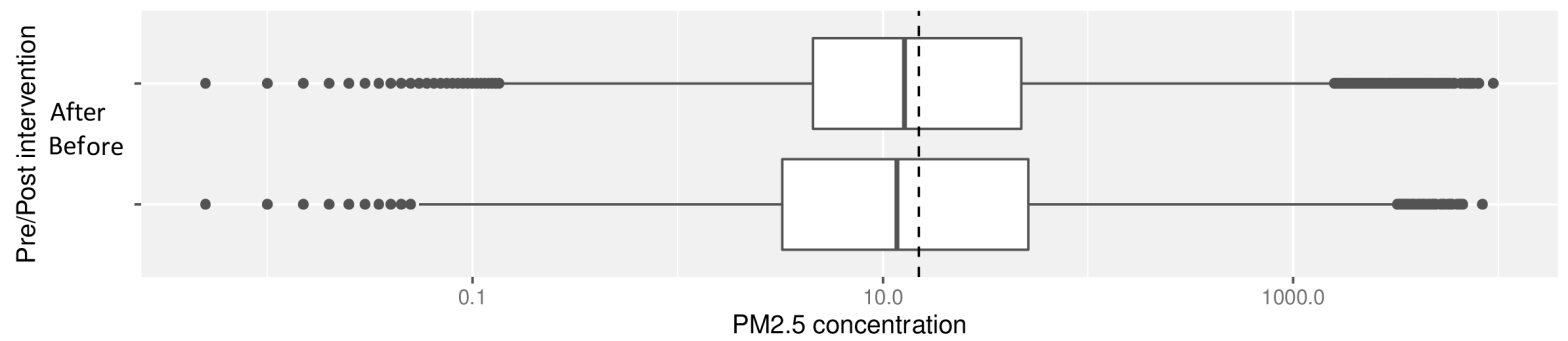
Box plot depicting overall median PM _2.5_ exposures before and after cookstove introduction, with PM _2.5_ concentrations plotted on a log scale. Dotted line indicates WHO-recommended 24-hour upper limit (PM _2.5_ concentration 15μg/m ^3^).

Median overall PM _2.5_ concentrations pre- and post-intervention were not significantly different: pre- and post-intervention medians and interquartile ranges (IQR) 11.8 μg/m ^3^ (IQR: 3.8 – 44.4) and 9.9 μg/m ^3^ (IQR: 2.2 – 46.5) respectively ( Figure 4, with dotted line to denote the WHO-recommended 24-hour upper limit (PM _2.5_ concentration 15μg/m ^3^) ^37^. Comparison of pre- and post- intervention medians grouped by participant number confirmed no significant difference between these concentrations (Wilcoxon V=95; p=0.71).

Matching activity data to traces, we found that median and interquartile range values during cooking activity before and after cookstove introduction were not significantly different (median and IQR for cooking-related concentrations pre- and post-intervention 79.4 μg/m ^3^ (IQR: 21.5 – 397.0) and 82.5 μg/m ^3^ (IQR: 37.3 – 307.0) respectively; V=86; p=1.00. Median and IQR concentrations were above WHO-recommended 24-hour upper limits throughout ( Figure 5a).

**Figure 5a.**
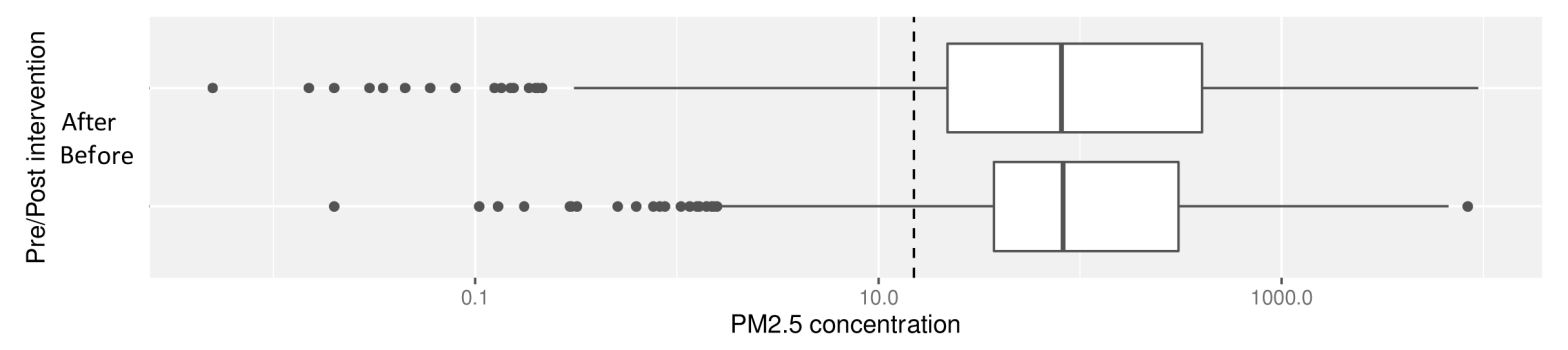
Box plot depicting cooking-related median PM _2.5_ exposures before and after cookstove introduction, with PM _2.5_ concentrations plotted on a log scale. Dotted line indicates WHO-recommended 24-hour upper limit (PM _2.5_ concentration 15μg/m ^3^).

During ‘baseline’ periods (no identified combustion activity), there was a statistically significant reduction in median PM _2.5_ concentrations after the introduction of stoves, from 8.2 μg/m ^3^ (IQR: 2.5 – 22.0) to 4.6 μg/m ^3^ (IQR: 1.0 – 12.6); V=141; p=0.01. This reduction brought more of the values below the WHO limits ( Figure 5b).

**Figure 5b.**
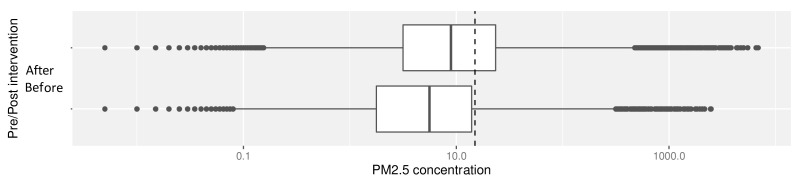
Box plot depicting ‘baseline’ median PM _2.5_ exposures before and after cookstove introduction, with PM _2.5_ concentrations plotted on a log scale. Dotted line indicates WHO-recommended 24-hour upper limit (PM _2.5_ concentration 15μg/m ^3^).

### Qualitative findings

**Cookstove use:** Observations throughout the village supported the finding from the monitoring sample of high cookstove uptake rates. On walking through the village, we frequently found people cooking on the cookstoves and there was good evidence of cookstove use at households we passed. Almost all the cookstoves were blackened with cooking smoke, and they were often covered in maize meal flour, suggesting habitual use. Notably, where previously three stones were to be seen in and around almost every household, these were now much less frequently seen. In some cases, the stones or bricks were seen to be discarded outside the yard. This was confirmed when raised in discussion with household members who, when asked where their three stone fires were, responded, *“palibe (there are none), we threw them away”*.

This finding, while frequent, was not universal, however. In discussion, a few residents mentioned using fires concurrently with their stoves if cooking had to be done quickly. In two households, women reported children (who were unused to the new stoves) using fires for cooking, and some women said that the stoves could not be used for very large amounts of food (for example when making *“thobwa”*, a fermented maize drink, and for cooking during special occasions such as weddings and funerals), although others’ accounts asserted the opposite view, confirming their use of the new stoves for these purposes.

One reason for not using the new stoves which was raised during several discussions was that firewood was sometimes in low supply. This related to the season, where there was little firewood to be found on the ground and this was sometimes damp or wet. In this situation, some residents bought small bags of charcoal, using this on charcoal stoves for the necessary household cooking. Charcoal cooking was infrequently seen however and was avoided by most in the village where possible, mainly due to its costs ^4^.

**Perceived benefits of cookstoves:** In response to questions around why participants liked and used the new cookstoves, there were a range of responses, of which the most common was that the stoves saved firewood. Participants used the same fuel as they would have used on their three stone fires – maize cobs (and at times maize stalks) as well as wood – and many claimed that their stoves *“uses less maize cobs or firewood than three stone fire”*. The stoves were thus felt to be cost saving. A fieldnote made during a conversation with a resident, which – when raised – resonated with many others, read:

*“(Female participant explained that) it saves firewood, so saves money too. Sometimes she has to buy firewood, money goes further when using (a firewood cookstove)”.*

Variations on this, which were also commonly stated, were that the fire in the stoves was shielded by the wind, and that the stove *“keeps the heat”*, thus allowing for ongoing cooking or bathwater warming, without the continuing use of fuel.

The second most commonly noted benefit of the stoves was faster cooking time *(“imafulumira”*), with some also noting the stove heating up more quickly than the time taken by a fire.

*“Our relish is now cooked in 10 minutes – previously, with a three stone fire, it would take until after 12"*

Fewer residents raised the issue of smoke in discussing benefits, indicating what this may not have been a priority. When asked specifically about smoke levels, opinions were split, with some feeling that the stoves produced more smoke, but others feeling that fires were worse. When discussing smoke levels, many people talked about fuel:

*“with wood, the firewood stove is better, even if using maize cobs, although with these there's more smoke than with wood”*

*“Wet wood is smoky at first, then it dries and is better – there’s no difference between the stove and three stone fire. I would still use the firewood stove with wet wood”*

It was noted that the benefit of not having to tend to the fire in the stove as much as a three stone fire (as it was protected from the wind) and being able to move the stove inside or outside, allowed them a degree of control over control their smoke exposures while cooking. This was supported by a quantitative finding of more cooking taking place outdoors in the post-intervention phase than pre-intervention ^29^.

**Perceived disadvantages of cookstoves:** The main issue raised with the cookstoves was that of breakage. We observed a number of stoves which had cracks in the sides already, although in most cases these stoves continued to be used. The cracks rarely prohibited the use of stoves but did mean that these participants refrained from using very large pots on the stoves, out of caution, and from moving them to different places.

We came across a few stoves in which, over time, cracks had progressed to significant breakage (and a piece of the stove was completely displaced). In one of these cases, the resident had bound wire around the cookstove rim to hold it together, allowing her to continue to use the stove. In the other cases, the stoves could no longer be used and were discarded, with residents in these households having reverted to the use of three stone fires. When asked about replacing the broken stoves, residents were positive, with most stating that they would pay between 1000 and 2000MK (approximately 1.20 – 2.50 USD): approximately the market price of the stoves. The extract from a conversation below illustrates many residents’ thoughts on replacing the stoves:

*“Me: Would you buy another? How much would you spend?*

*Female resident: Yes. 1000, 1500, 2000 kwacha. "Anthu azolowera" (people have now become used to the stoves)”*

The main concern for most was that the stoves were not available for sale in the area, and that transport to the nearest market where they could be purchased would make their replacement unaffordable.

## Discussion

Four to six months after the introduction of locally made clay stoves in the village, the new stoves were being used in most households, and for most of the cooking and bathwater warming activity. In the sample of participants involved in personal exposure monitoring, there was no change in PM _2.5_ exposures with the introduction of the new stoves, although ‘baseline’ exposures – in the absence of specific combustion activity – were lower post-intervention. Qualitative data revealed a widespread approval of the stoves amongst residents, with the main reason stated being their more efficient use of fuel. Cracking of the stoves with use was a key issue raised, and is a relatively commonly reported issue with these basic stoves, often related to quality of clay or manufacturing processes ^38, 39^, although residents seemed keen to replace the stoves, should they be available for sale.

The widespread use of the new stoves was apparent in both the time-activity data collected alongside air quality monitoring, and in participant observation data, with both sources clearly indicating a replacement of previous cooking methods with the new stoves. This is notable, given the prevalence of ‘stacking’ (combined use of multiple cooking modalities, old and new, rather than replacement) following the introduction of ‘improved’ cooking technologies ^40– 43^. This relates to the reasons for continued use of traditional stoves, which vary but include limitations of newly introduced technologies, need for concurrent cooking on multiple stoves, and fuel access and cost, as well as (less commonly) different context-specific cooking needs ^40, 41, 43, 44^. Participants in this study raised some of these issues, namely that of using multiple devices concurrently, although when asked they stated that they would use two stoves if they were available. Issues with fuel access were also sometimes raised, in keeping with previous findings around resource limitations in this setting ^4^.

In spite of the widespread cookstove use amongst the cohort, there was no difference in individuals’ PM _2.5_ exposures, either overall or during cooking periods, after introduction of the stoves. This is perhaps unsurprising given the lack of clear evidence of exposure reduction with these basic cookstove types, compared with traditional cooking fires ^45^. Participants’ observations of faster cooking time and less need to tend the fire when cooking on the new stoves signpost the potential for reductions in personal emissions on a larger scale – although this was not seen in our small sample of participants. Our finding of reductions in ‘background’ exposure (during non-cooking time) could reflect a previously reported greater reduction in smouldering emissions ^46^ and, given the decrease further below WHO-recommended thresholds, may be an encouraging direction of change from traditional stoves.

These outcomes could be framed in terms of implementation science frameworks such as the RE-AIM framework ^47, 48^ with statements relating to the high levels of ‘adoption’ and ‘reach’, poorer ‘effectiveness’ outcomes – judged in terms of researcher plans to reduce air pollution – and thoughts around ensuring ‘maintenance’ of the intervention in the longer term. This approach, with assessments made only in respect to researchers’ predetermined aims and outcomes, was not the aim of the study however. Our ethnographic work allowed insights into participants’ lived experiences, enriching the evaluation and helping us to understand it’s value from a range of perspectives.

In qualitative discussions, residents’ main comments on the new stoves related not to ‘smoke’, but to perceived reductions in fuel use compared with three stone fires which they replaced, reflecting improvements in burning efficiency. This efficiency benefit is reported in the literature, although improvements with basic stoves tend to be modest compared with more advanced cookstoves ^45, 49, 50^. The positive reception to the stoves seen in our study echo community responses to the introduction of the Jambar (another simple biomass stove with efficiency benefits) in rural Senegal ^51, 52^. Researchers Jeuland et al. note that “reducing firewood and charcoal consumption are important objectives in themselves – both from environmental and poverty alleviation perspectives” ^22^. This is particularly relevant in a setting such as rural Malawi in which many residents’ lives are shaped by severe economic scarcity, and where access to food, and fuel on which to cook daily meals, are prime concerns ^4^.

Researchers conducting the trial in Senegal and others have noted that participants’ willingness to pay for new stoves was high despite their initial free provision, and that their widespread provision to all community members positively their uptake ^16, 52^. Findings of the current study agree with this, in that positive reports of the stoves were far more forthcoming after their introduction across the village than before the intervention from the few households which owned the stoves ^4^. This village-level approach is also important in view of the shared nature of air pollution, with widespread uptake of cleaner technologies required to accrue air quality benefits ^53, 54^.

Strengths of our study lie in the combined use of qualitative observations and quantitative data collection to allow a realist evaluation of the intervention – delivered on a whole-village level – in its intended context, and activity matched exposure data. We acknowledge that our study had limitations, namely the small sample of participants involved in the quantitative ‘air quality monitoring’ component, and the slight difference in timing of pre- and post-intervention phases resulting in the widespread use of maize cobs as fuel in the post-intervention phase. Outcomes of air quality monitoring were broadly in keeping with expectations however, adding evidence around potential reductions in exposures during the ‘smouldering’ phase. These findings should be further explored with larger scale monitoring studies, using techniques such as those we have employed to decouple cooking- and non-cooking related exposures.

In conclusion, whilst there were no cooking-associated reductions in PM _2.5_ exposure after introduction of the cookstoves, the stoves were welcomed and widely used by residents across the village. Residents valued the efficiency and fast cooking of these stoves – responding to key local priorities – and these factors as well as less need to tend the fire and the possibility of moving the site of cooking also hold the potential for small reductions in population-level exposure.

Whilst significant improvements in air quality will require a more comprehensive approach ^24, 55, 56^, accessible cooking solutions such as these stoves with the potential to meet communities’ immediate needs represent a valued interim alternative to cooking on open fires. Scale up of production and distribution to allow more households to replace their stoves once broken, or even schemes to support local production, are required to allow more communities access to these simple technologies.

## Data availability

### Underlying data

Harvard Dataverse: Comparative pre-post PM2.5 data, https://doi.org/10.7910/DVN/PNYOTX. ^29^

This project contains the following underlying data:

- ppSet2.Raqm files.Rdata
- ppSetT.Raqm files.Rdata

### Extended data

Harvard Dataverse: Comparative pre-post PM2.5 data, https://doi.org/10.7910/DVN/PNYOTX.

This project contains the following extended data:

- MM paper Supplement.docx

Data are available under the terms of the Creative Commons Zero "No rights reserved" data waiver (CC0 1.0 Public domain dedication).

## Reflexivity statement

The following reflexivity statement details key elements of the research partnership, conduct and reporting of the work presented above, in the hope that transparency with regard to transnational research practices will lay a foundation for more equitable ways of conducting collaborative research across the academic system.

**Table:** 

**Study conceptualization**	**1. How does this study address local research and policy priorities?** Air pollution is a global health priority. Malawi is a low-income country with high levels of air pollution and consequent morbidity. Cooking using solid fuels is thought to be a key contributor to airborne pollutant exposure in rural populations. Our interventional study – informed by an in-depth ethnographic account of air pollution (or ‘smoke’) in the setting – involved the introduction of a locally made cookstove in an effort to reduce individuals’ exposures while also considering residents’ other priorities relating to their health and wellbeing.
	**2. How were local researchers involved in study design?** The research assistant (HS) for this study is a local social scientist based in Malawi with previous experience doing research in this area. He was involved with study design and data collection and ensured that approaches and methods were context-appropriate throughout. The fieldworker (DM) is a resident in the village in which the study is based and contributed perspectives in study design and implementation as well as optimising linkages with the community throughout the wider study.
**Research management**	**3. How has funding been used to support the local research team(s)?** Part of the research funding was used to provide salaries for local researchers – as above – and staff involved in the broader research grant including research governance and grants management.
**Data acquisition and** **analysis**	**4. How are research staff who conducted data collection acknowledged?** The research assistant and fieldworker worked with the main researcher on data collection, and the research assistant also supported data management activities. Both are authors of this paper with their specific contributions acknowledged appropriately.
	**5. How have members of the research partnership been provided with access to study data?** Study data is archived at Malawi Liverpool Wellcome Trust. Local researchers have direct access to the data.
	**6. How were data used to develop analytical skills within the partnership?** The PhD researcher (SS) supported the research assistant in quantitative data management as well as analysis of quantitative and qualitative data, helping to develop these skills further.
**Data interpretation**	**7. How have research partners collaborated in interpreting study data?** Data interpretation involved discussions around analytical decisions and methods, which incorporated various members of the team (based in Malawi and the UK)
**Drafting and revising** **for intellectual content**	**8. How were research partners supported to develop writing skills?** The lead author of this paper is a doctoral candidate. She led in writing the paper, with reflective input and advice from all partners.
	**9. How will research products be shared to address local needs?** Preliminary findings have been shared within the village at dissemination events. Earlier quantitative data have been presented at local research dissemination conferences and within the research institution (MLW), and these forms of sharing will continue with the present data. This manuscript will be made available to the wider global scientific community for discussion and development of the findings.
**Authorship**	**10. How is the leadership, contribution and ownership of this work by LMIC researchers** **recognised within the authorship?** Please refer to the section on “Authors’ contribution” in the manuscript. Each author’s role is described including researchers from LMICs.
	**11. How have early career researchers across the partnership been included within the** **authorship team?** Please refer to question 8 above regarding leadership of the project. The study also incorporated a junior researcher in the LMIC setting as research assistant and a local fieldworker who had not previously had any research involvement, all included as authors.
	**12. How has gender balance been addressed within the authorship?** The research lead (whose doctoral work is represented here) is female, as are 3/7 of the authors, with representation from both local LMIC and HIC settings. Contributions to the study are acknowledged in the “Authors’ contribution” section of the manuscript
**Training**	**13. How has the project contributed to training of LMIC researchers?** The research assistant (HS) has been involved in the research process throughout, developing key skills, and has been supported in successfully applying for a Masters’ scholarship in Global Health research. Involvement of the local fieldworker (DM) constituted her first experience of research participation. Both have significantly contributed to the project and are recognised accordingly in the authorship. These experiences will lay the foundation for further academic career development.
**Infrastructure**	**14. How has the project contributed to improvements in local infrastructure?** Whilst this is a small scale study, the project team have strived to support constructive engagement between the village community and the research institution throughout. Stoves were provided to all households as part of the study and links have been made with the local provider to enable residents to purchase replacement stoves in the future. Work is also underway to create a nursery/health centre in the village to express thanks to residents for their involvement and to provide continuity of employment for the local fieldworker. With reference to question 3 above, research governance, ethics and grant management systems of the local implementing partner (MLW) were supported through this grant.
**Governance**	**15. What safeguarding procedures were used to protect local study participants and** **researchers?** The local ethics body and LSTM research ethics committee reviewed and approved the study protocol ensuring that both participants and researchers are protected throughout the study. Among other considerations, participants provided informed consent prior to their participation and, specifically, a named safeguarding lead (SS) was in place throughout, with various avenues of contact for participants to report any concerns, and structures for appropriate referral of any such reports.
