# Supplementary material for: “We threw away the stones”: a mixed method evaluation of a simple cookstove intervention in Malawi
Source: Wellcome Open Res. 2022 Jun 10;7:52. Originally published 2022 Feb 14. [Version 3] doi: 10.12688/wellcomeopenres.17544.3 (PMC8933645; doi:10.12688/wellcomeopenres.17544.3)
Supplement: Peer review history

# Peer review: Reviewer response for version 2

**DOI:** 10.21956/wellcomeopenres.19686.r49276

**Published:** 2022-03-18

**Authors:** Marc Jeuland

**Affiliations:** 1 Sanford School of Public Policy, Duke Global Health Institute, Duke University, Durham, NC, USA

Thank you for the revisions made in response to my comments. I found them to be responsive and on point.

Is the work clearly and accurately presented and does it cite the current literature?

Yes

If applicable, is the statistical analysis and its interpretation appropriate?

Partly

Are all the source data underlying the results available to ensure full reproducibility?

Yes

Is the study design appropriate and is the work technically sound?

Yes

Are the conclusions drawn adequately supported by the results?

Partly

Are sufficient details of methods and analysis provided to allow replication by others?

Yes

Reviewer Expertise:

Environmental health, energy access, economics

I confirm that I have read this submission and believe that I have an appropriate level of expertise to confirm that it is of an acceptable scientific standard.

# Peer review: Reviewer response for version 1

**DOI:** 10.21956/wellcomeopenres.19400.r48694

**Published:** 2022-03-09

**Authors:** Deborah Stanistreet

**Affiliations:** 1 Department of Epidemiology and Public Health Medicine, Royal College of Surgeons in Ireland, Dublin, Ireland

There is a dearth of mixed methods papers in the HAP field that integrate quantitative findings with the qualitative data that explores the perspectives of the cookstove user and this is a well written and interesting paper that addresses just that issue.

 The Mbaula is indeed a cheap ‘improved’ cookstove in Malawi, one of the only improved stoves that communities can afford. Many communities have benefited from acquiring one over the three stone fire for a number of reasons, but for a number of reasons, the ability of the stove to reduce emissions had varied between studies.  From our (soon to be published) scoping review it is also the only improved cookstove on the market which is cheap enough and accessible to the poorest populations in Malawi and therefore exploring community perceptions alongside HAP reduction is very useful.

 This particular study found no significant difference in PM2.5 levels between the three stone fire and the Mbaula, but it may be worth also examining the evidence regarding whether the Mbaula can reduce HAP in a laboratory setting through the Clean Cookstoves Catalogue and to discuss some of the differences in performance between laboratory and field setting use and how this might be addressed.

http://catalog.cleancookstoves.org/stoves/385

 Of particular interest is the discussion about the community views of the stoves and how this can potentially impact uptake and sustained use, as historically, there has been a greater focus on the technical aspects of the stove with less emphasis given to community preference. Might it be feasible to recommend the adoption of other behaviours that would reduce HAP alongside the Mbaula such as improved ventilation, facilities to dry wood, etc. as a means of reducing emissions further?

Is the work clearly and accurately presented and does it cite the current literature?

Yes

If applicable, is the statistical analysis and its interpretation appropriate?

Yes

Are all the source data underlying the results available to ensure full reproducibility?

Yes

Is the study design appropriate and is the work technically sound?

Yes

Are the conclusions drawn adequately supported by the results?

Yes

Are sufficient details of methods and analysis provided to allow replication by others?

Yes

Reviewer Expertise:

public health

I confirm that I have read this submission and believe that I have an appropriate level of expertise to confirm that it is of an acceptable scientific standard.

# Response

**Published:** 2022-03-16

**Authors:** Sepeedeh Saleh

**Affiliations:** Liverpool School of Tropical Medicine, UK

Many thanks to Dr Stanistreet for this review and the interesting and very relevant comments. We eagerly anticipate reading the upcoming scoping review mentioned, and we agree with the suggestion of the need for further evidence around the stove’s performance in different environments, in view of its wide accessibility in very low-income settings.

 We are grateful for the comments on the possibility of other ‘behavioural’ elements influencing overall exposures. A paper describing details of individual exposures in the village (pre-intervention), which provides evidence on the roles of fuel type and ventilation in shaping exposures, is currently under review, and we look forward to contributing further to these ongoing discussions around determinants of exposure in the rural African setting.

# Peer review: Reviewer response for version 1

**DOI:** 10.21956/wellcomeopenres.19400.r48698

**Published:** 2022-03-07

**Authors:** Marc Jeuland

**Affiliations:** 1 Sanford School of Public Policy, Duke Global Health Institute, Duke University, Durham, NC, USA

**Review of ‘“We threw away the stones”: a mixed method evaluation of a simple cookstove intervention in Malawi’**

**Summary**

 This is an interesting mixed methods study that explores adoption and use of simple biomass improved cookstoves in a rural village in Malawi. The authors conducted ethnographic research alongside measurement of exposures and time spent cooking to obtain richer insights on fuel and stove use practices and perceptions. The real strength of the study, in my opinion, is the ethnographic work, and I feel that the exposure results are somewhat overemphasized given the lack of a theoretical basis for expecting much exposure reduction. In trying to explain the exposure results (or lack thereof) the authors mostly speculate, without really nailing things down. More general and specific comments follow below.

**General comments**

 I generally like this paper and the point it is making, but I think that the central role afforded to exposure actually detracts. It seems like the paper is almost apologetic about the lack of significant improvements in exposure. In so doing, it ironically elevates that issue and puts it on an equal footing with the more interesting ethnographic aspects and insights. I would urge the authors to re-equilibrate the discussion to be more consistent with their points about livelihoods.

*Introduction, the sentence:*

- “Centring local perspectives in this way, as well as constituting arguably the ‘right’ approach to global health problems, can optimise the suitability and sustainability of any subsequent solutions”. This phrase is needlessly ambiguous. First, I am not sure what the authors mean by the “right” approach. I also think “optimize the suitability and sustainability” is too strong a phrase. Just centering local perspectives does not guarantee optimizing these rather difficult objectives. Moreover, local perspectives can actually be in direct conflict with sustainability, if we are considering environmental sustainability, for example.

*Study setting:*

- I appreciate that the paper reference previous studies in this particular location, but a reader of this article would like to know a bit about the site without having to refer to those prior studies. What are the main economic livelihoods activities in this location? Are there particularities of cooking and fuel collection activities there that are worth highlighting? Etc. This is especially valuable for understanding the context and broader implications of this research.

*Study design and intervention:*

- “Although they were known about by many in the village, few households already owned one of these firewood cookstoves.” This is a very important sentence, that raises many questions. If known about, why didn’t more households have these stoves? What had been the experiences with the stoves? Surely this would influence how people responded to the intervention. I noted that the authors discuss this point a bit on p.9 when emphasizing the importance of a “village-level” approach, but more background and discussion would be helpful about prior experiences and impressions.

*Data collection:*

- It would be useful to understand seasonal aspects of data collection. I gather from the manuscript that the pre- and post-intervention observations were 3 months apart. Were these in significantly different seasons? Also, what was the season for the the ten week period of observations, and how does this relate to behaviors and insights obtained? (Note that this comes up on p.6 as an explanation for changed fuel use, but a reader wants to know such details much earlier).

*Results:*

- Can the authors explain more why “combustion hours” increased post intervention? (p.5). Is this likely related to the intervention or due to some other time-varying phenomenon that was correlated with the intervention?
- Figure 4 and the statement about medians pre and post intervention do not seem to match. Am I misreading the plot?
- Did any households purchase firewood (not just charcoal)? My experience in Malawi tells me that this can be common in some areas, especially in the rainy season when firewood is wet. More details would be helpful here, including more explanation of the observation on p.7 that “This related to the season, where there was little firewood to be found on the ground and this was sometimes damp or wet.”
- The authors say “Fewer residents raised the issue of smoke in discussing benefits, indicating what this may not have been a priority.” First of all, the sentence is not grammatically correct. But more importantly, isn’t the more logical explanation that this basic stove does not really reduce emissions? Concluding that this is not a priority is a stretch.
- How should we interpret the reduction in “baseline” exposures? (there is speculation of less smouldering, but this seems unlikely to me unless it was observed – in any case, evidence is not presented that this is the key mechanism at work). Also, throughout, I find the term baseline confusing, because it suggests pre-intervention, but the authors instead mean background exposures. I suggest just saying “background” exposures. Is the change due to seasonality – since dust and ambient pollution could be lower in the post-intervention period? Or could it be about different time use patterns due to season (more time in the fields, where air is cleaner)?
- Findings of significant breakage just a few months after intervention are concerning, but the authors don’t really reflect much on this issue and how villagers were thinking about it.

*Discussion:*

- “Four to six months”. I thought the observations began after 3 months. Or should it be 4 months?
- “Residents valued the efficiency and fast cooking of these stoves – responding to key local priorities – and these factors as well as less need to tend the fire and the possibility of moving the site of cooking also hold the potential for small reductions in population-level exposure.” The second part of this sentence goes too far, given the results of the study. And again, it almost devalues the first part of the sentence, apologizing for the lack of exposure improvements.

**Specific comments**

*Introduction:*

- “In Malawi, which is largely rural, air pollution is a persisting problem”. Do you mean “persistent”?
- “Thus, while individual household interventions will not be sufficient to achieve clinically impactful reductions in PM2.5 there may be benefits to community-level adoption of locally relevant cleaner stove types in low-income settings such as Malawi.” Is the “community-level” qualifier needed here? Wouldn’t there even perhaps be benefits from individual adoption? (e.g., fuel and time savings)

*Data Collection:*

- “Discussions were often based around cooking activities, partly because this was the activity families were most often engaged in when spending time around the household.” It would be nice to define “cooking activities”. In particular, I find it hard to believe that cooking was the activity with the most time spent, unless cooking is defined rather broadly to include cleaning, firewood collection and preparation, etc. Some more precision here would be helpful. Cooking hours themselves are likely significant, but rarely the dominant form of time use in settings such as this one.

*Discussion:*

- “Researchers conducting the trial in Senegal and others have noted that participants’ willingness to pay for new stoves was high despite their initial free provision, and that their widespread provision to all community members positively their uptake”. There’s a word missing in the latter part of the sentence.

Is the work clearly and accurately presented and does it cite the current literature?

Yes

If applicable, is the statistical analysis and its interpretation appropriate?

Partly

Are all the source data underlying the results available to ensure full reproducibility?

Yes

Is the study design appropriate and is the work technically sound?

Yes

Are the conclusions drawn adequately supported by the results?

Partly

Are sufficient details of methods and analysis provided to allow replication by others?

Yes

Reviewer Expertise:

Environmental health, energy access, economics

I confirm that I have read this submission and believe that I have an appropriate level of expertise to state that I do not consider it to be of an acceptable scientific standard, for reasons outlined above.

# Response

**Published:** 2022-03-15

**Authors:** Sepeedeh Saleh

**Affiliations:** Liverpool School of Tropical Medicine, UK

Dear Prof Jeuland

 Many thanks for your review of this paper, which has now been revised in response to the comments.

 We have reproduced and addressed individual comments below (reviewer comments in italics).

*Introduction, the sentence:*

- *“Centring local perspectives in this way, as well as constituting arguably the ‘right’ approach to global health problems, can optimise the suitability and sustainability of any subsequent solutions”. This phrase is needlessly ambiguous. First, I am not sure what the authors mean by the “right” approach. I also think “optimize the suitability and sustainability” is too strong a phrase. Just centering local perspectives does not guarantee optimizing these rather difficult objectives. Moreover, local perspectives can actually be in direct conflict with sustainability, if we are considering environmental sustainability, for example.*

Thank you for this comment. The section in question has now been rewritten to improve the clarity and accuracy of the message.

*Study setting:*

- *I appreciate that the paper reference previous studies in this particular location, but a reader of this article would like to know a bit about the site without having to refer to those prior studies. What are the main economic livelihoods activities in this location? Are there particularities of cooking and fuel collection activities there that are worth highlighting? Etc. This is especially valuable for understanding the context and broader implications of this research.*

Thank you for noting this. Appropriate detail has now been added in the text under ‘Study setting and population’.

*Study design and intervention:*

- *“Although they were known about by many in the village, few households already owned one of these firewood cookstoves.” This is a very important sentence, that raises many questions. If known about, why didn’t more households have these stoves? What had been the experiences with the stoves? Surely this would influence how people responded to the intervention. I noted that the authors discuss this point a bit on p.9 when emphasizing the importance of a “village-level” approach, but more background and discussion would be helpful about prior experiences and impressions.*

Many thanks, Prof Jeuland, for pointing this out. Relevant information has been added in the text. The key issue here is that we came across only two households which had these (donated) cookstoves so there was little scope for wider discussion of residents’ experiences with these.

*Data collection:*

- *It would be useful to understand seasonal aspects of data collection. I gather from the manuscript that the pre- and post-intervention observations were 3 months apart. Were these in significantly different seasons? Also, what was the season for the ten week period of observations, and how does this relate to behaviors and insights obtained? (Note that this comes up on p.6 as an explanation for changed fuel use, but a reader wants to know such details much earlier).*

Thank you for picking up this valuable area of discussion. There was a lack of clarity in the paper as it was written, in relation to study timescales. This has now been revised (under the ‘Study design and intervention’ and ‘Data collection’ sections). The pre-intervention and post-intervention data collection periods actually took place during similar months over successive years. Most seasonal aspects should therefore be comparable between the two periods. A key difference here relates to the timing of the harvest, which fell during the post-intervention monitoring period (but not during the pre-intervention period of monitoring). This had repercussions on fuel use, as described in the paper.

*Results:*

- *Can the authors explain more why “combustion hours” increased post intervention? (p.5). Is this likely related to the intervention or due to some other time-varying phenomenon that was correlated with the intervention?*

Thank you for raising this question. Our extended period of qualitative observation gave us insights into the likely reason for this, linked to the burning of maize cobs as fuel, which was inefficient and therefore extended cooking times, in comparison to firewood. This had been added to the text (under ‘Cooking characteristics’).

  

- *Figure 4 and the statement about medians pre and post intervention do not seem to match. Am I misreading the plot?*

We are grateful for this astute observation. The data as presented in figure 4 reflected the original values with some zero values removed by the system, due to the log scale. This was the cause of the incoherent figure. All three box plots have now been replaced, with the use of corrected values to ensure the full data are presented. The text in the relevant sections has also been revised to reflect this.

  

- *Did any households purchase firewood (not just charcoal)? My experience in Malawi tells me that this can be common in some areas, especially in the rainy season when firewood is wet. More details would be helpful here, including more explanation of the observation on p.7 that “This related to the season, where there was little firewood to be found on the ground and this was sometimes damp or wet.”*

Thank you for this observation. More information on fuel purchase has now been added, under ‘Study setting and population’, as well as the section described above. In relation to the specific question raised, we observed that village residents rarely bought firewood (this tended to be done only if households came across more money than they usually had access to) – mainly due to the costs of purchasing (large) bundles of firewood. Charcoal – being more commonly sold in small bags – was the more usual purchased fuel of choice where firewood was not freely available.

  

- *The authors say “Fewer residents raised the issue of smoke in discussing benefits, indicating what this may not have been a priority.” First of all, the sentence is not grammatically correct. But more importantly, isn’t the more logical explanation that this basic stove does not really reduce emissions? Concluding that this is not a priority is a stretch.*

Many thanks for noting this. The relevant sentence has been rewritten to better reflect the subsequent discussion around residents’ perceptions on the stoves and smoke levels.


* *

- *How should we interpret the reduction in “baseline” exposures? (there is speculation of less smouldering, but this seems unlikely to me unless it was observed 10/21 & 13* */21* *– in any case, evidence is not presented that this is the key mechanism at work). Also, throughout, I find the term baseline confusing, because it suggests pre-intervention, but the authors instead mean background exposures. I suggest just saying “background” exposures. Is the change due to seasonality – since dust and ambient pollution could be lower in the post-intervention period? Or could it be about different time use patterns due to season (more time in the fields, where air is cleaner)?*

We are grateful, Prof Jeuland, for your raising of this question and for noting the confusion around the use of the word ‘baseline’. The use of ‘baseline’ has been reviewed throughout the paper, and changes made, in line with reviewer suggestions, to avoid confusion.

 Regarding the question of the reduction in background exposures, the suggestion of smouldering is a proposed cause, but one supported by our extended observations in the field, both over a period of nine months before any intervention and following the introduction of the stoves. We regularly witnessed (and experienced) fires smouldering for periods of time following one cooking episode, and between episodes of cooking or bathwater warming, while household members served food, ate, and relaxed in the yard or veranda. During our time in and around households after the intervention, we noted that this was barely ever the case with cookstoves, and thus could be a likely mechanism for the reduced background exposures post-intervention. These details have been added to the manuscript in the relevant sections.

 As discussed above, there were few seasonal differences between the pre- and post-intervention periods, as these periods covered similar months, both in the second half of the rainy season.

  

- *Findings of significant breakage just a few months after intervention are concerning, but the authors don’t really reflect much on this issue and how villagers were thinking about it.*

Thank you for this point. Whilst the cracks in most cases did not constitute ‘significant breakage’ (most cracks were cosmetic, as described in the text, not interfering with cooking function), we agree that this had potential repercussions for intervention sustainability. This could be compounded by the issue of the stoves not being sold in or close to the village, and subsequent added costs of accessing now stoves. Unfortunately, these ongoing issues were too expansive to include in the current paper, whose scope was already felt to be quite broad. We agree that sustainability of this intervention over time will be an important subject to assess in future publications.

*Discussion:*

- *“Four to six months”. I thought the observations began after 3 months. Or should it be 4 months?*

Many thanks again for noting the confusion with regard to timescales in this paper. The ‘Study design and intervention’ and ‘Data collection’ sections have been revised to clarify these issues, as has this sentence.

  

- *“Residents valued the efficiency and fast cooking of these stoves – responding to key local priorities – and these factors as well as less need to tend the fire and the possibility of moving the site of cooking also hold the potential for small reductions in population-level exposure.” The second part of this sentence goes too far, given the results of the study. And again, it almost devalues the first part of the sentence, apologizing for the lack of exposure improvements.*

We are grateful for this comment and agree with your point, Prof Jeuland. The sentence in question has been altered accordingly.

***Specific comments***

*Introduction:*

- *“In Malawi, which is largely rural, air pollution is a persisting problem”. Do you mean “persistent”?*

Thank you for this suggestion. The word has been changed to ‘persistent’.

  

- *“Thus, while individual household interventions will not be sufficient to achieve clinically impactful reductions in PM2.5 there may be benefits to community-level adoption of locally relevant cleaner stove types in low-income settings such as Malawi.” Is the “community-level” qualifier needed here? Wouldn’t there even perhaps be benefits from individual adoption? (e.g., fuel and time savings)*

Thanks again for this comment, with which we agree. We have removed the qualifier and slightly altered the sentence to reflect this point.

*Data Collection:*

- *“Discussions were often based around cooking activities, partly because this was the activity families were most often engaged in when spending time around the household.” It would be nice to define “cooking activities”. In particular, I find it hard to believe that cooking was the activity with the most time spent, unless cooking is defined rather broadly to include cleaning, firewood collection and preparation, etc. Some more precision here would be helpful. Cooking hours themselves are likely significant, but rarely the dominant form of time use in settings such as this one.*

We are grateful for this interesting observation. Our meaning here was that, in the post-intervention period, during which we mostly spent time and around households, the activities we observed (and at times took part in) around the household were largely related to cooking. This is in contrast to wider daily activities such as working on farms, bringing water from the well, and going to the shops, which took place away from the household. We also agree that the phrase ‘cooking and related activities’ better reflects our meaning here. We have revised the text to better explain this.

*Discussion:*

- *“Researchers conducting the trial in Senegal and others have noted that participants’ willingness to pay for new stoves was high despite their initial free provision, and that their widespread provision to all community members positively their uptake”. There’s a word missing in the latter part of the sentence.*

Many thanks for noting this omission. The missing word has now been added.

 Once again, we thank you for your appreciation of the paper content and for the thorough and insightful review, which has much improved the paper.

 Kind regards

 Dr Sepeedeh Saleh MBChB, MPH, MFPH, DTMH, Wellcome Trust Clinical PhD Fellow,

 on behalf of the ‘PAMODZI’ project team
